# Pairwise machine learning-based automatic diagnostic platform utilizing CT images and clinical information for predicting radiotherapy locoregional recurrence in elderly esophageal cancer patients

**DOI:** 10.1007/s00261-024-04377-7

**Published:** 2024-06-04

**Authors:** An-du Zhang, Qing-lei Shi, Hong-tao Zhang, Wen-han Duan, Yang Li, Li Ruan, Yi-fan Han, Zhi-kun Liu, Hao-feng Li, Jia-shun Xiao, Gao-feng Shi, Xiang Wan, Ren-zhi Wang

**Affiliations:** 1 Department of Radiotherapy, Hebei Medical University Fourth Affiliated Hospital and Hebei Provincial Tumor Hospital, 12 Jiankang Road, Shijiazhuang, Hebei 050011 People’s Republic of China; 2 School of Medicine, Chinese University of Hong Kong (Shenzhen), No. 2001, Longxiang Avenue, Longgang District, Shenzhen, 518172 People’s Republic of China; 3 Medical Big Data Laboratory, Shenzhen Research Institute of Big Data, Daoyuan Building, No. 2001, Longxiang Avenue, Longgang District, Shenzhen, 518172 People’s Republic of China; 4 Department of Oncology, Hebei General Hospital, NO. 348 Heping West Road, Xinhua District, Shijiazhuang, Hebei 050051 People’s Republic of China; 5 School of Computer Science and Engineering, Beihang University, No. 37 Xueyuan Road, Haidian District, Beijing, 100191 People’s Republic of China

**Keywords:** Carcinoma, Squamous cell, Esophageal neoplasms, Radiotherapy, Locoregional recurrence, Machine learning

## Abstract

**Objective:**

To investigate the feasibility and accuracy of predicting locoregional recurrence (LR) in elderly patients with esophageal squamous cell cancer (ESCC) who underwent radical radiotherapy using a pairwise machine learning algorithm.

**Methods:**

The 130 datasets enrolled were randomly divided into a training set and a testing set in a 7:3 ratio. Clinical factors were included and radiomics features were extracted from pretreatment CT scans using pyradiomics-based software, and a pairwise naive Bayes (NB) model was developed. The performance of the model was evaluated using receiver operating characteristic (ROC) curves and decision curve analysis (DCA). To facilitate practical application, we attempted to construct an automated esophageal cancer diagnosis system based on trained models.

**Results:**

To the follow-up date, 64 patients (49.23%) had experienced LR. Ten radiomics features and two clinical factors were selected for modeling. The model demonstrated good prediction performance, with area under the ROC curve of 0.903 (0.829–0.958) for the training cohort and 0.944 (0.849–1.000) for the testing cohort. The corresponding accuracies were 0.852 and 0.914, respectively. Calibration curves showed good agreement, and DCA curve confirmed the clinical validity of the model. The model accurately predicted LR in elderly patients, with a positive predictive value of 85.71% for the testing cohort.

**Conclusions:**

The pairwise NB model, based on pre-treatment enhanced chest CT-based radiomics and clinical factors, can accurately predict LR in elderly patients with ESCC. The esophageal cancer automated diagnostic system embedded with the pairwise NB model holds significant potential for application in clinical practice.

**Supplementary Information:**

The online version contains supplementary material available at 10.1007/s00261-024-04377-7.

## Introduction

Esophageal cancer (EC) is a prevalent malignancy within the digestive system, ranking seventh globally in terms of incidence and sixth in mortality [1]. In China, the incidence and mortality rates of esophageal cancer rank sixth and fourth, respectively, displaying a higher trend compared to the global scenario [2]. The aging population and increased life expectancy have contributed to a rise in elderly EC patients. Notably, individuals aged 75 and above constitute approximately 15–20% of the EC patient population in China [2]. Owing to the presence of comorbidities and diminished organ functional reserve, elderly patients face limitations in their ability to tolerate intensive treatments when compared to the nonelderly, which may contribute to inferior outcomes [3]. Radiotherapy (RT) serves as the primary treatment modality for elderly patients with EC, particularly for those who are ineligible for surgery [4]. Nevertheless, more than 50% of patients undergoing standard dose chemoradiotherapy (CRT) eventually experience local regional recurrence or distant metastases, resulting in disease-related mortality [3, 5]. The amalgamation of radiomics, coupled with various classifiers, facilitates a comprehensive evaluation of temporal and spatial heterogeneities using quantitative analysis applied to sequential data. This approach has promising potential in predicting treatment responses across various cancer types [6–8]. Notably, several studies have highlighted the predictive and prognostic value of radiomics in EC patients treated with CRT [9–11]. To our knowledge, there is a lack of radiomics studies utilizing machine learning techniques to predict locoregional recurrence (LR) in elderly patients with ESCC who have undergone RT. In this investigation, we developed a pairwise machine learning modeling method, leveraging metric learning and employing the open-source project “FeAture Explorer” [12] (available at https://github.com/salan668/FAE), to enhance prediction accuracy. We developed an effective pairwise naïve Bayes (NB) model through an optimized modeling process.

The primary objective of this study was to assess the feasibility of the pairwise machine learning model in predicting LR following RT in elderly patients diagnosed with esophageal squamous cell carcinoma (ESCC), using clinical factors and quantitative radiomics features extracted from pretreatment contrast-enhancement CT scans. By providing accurate and early predictions to assist physicians in making optimal therapeutic decisions, facilitating the adjustment of treatment plans and timely interventions. Finally, to facilitate practical application, an automated esophageal cancer diagnosis system based on trained models were developed.

## Materials and methods

### Dataset and preparation

#### Inclusion criteria and study population

The ethical approval for this study was obtained from the ethics committee of the Fourth Affiliated Hospital of Hebei Medical University. According to the definition of the World Health Organization, we employed an age cutoff of 75 years to classify patients as elderly individuals. Because ESCC accounted for almost 90% of all EC instances in China [13], we limited our analysis to patients with ESCC diagnosed by pathology. The following selection criteria were applied: (1) Age 75 or above. (2) Eastern Cooperative Oncology Group performance status (ECOG PS) of 2 or less. (3) No prior history of cancer. (4) Radiation therapy was administered to each patient for the first time. (5) Absence of distant organ metastasis except for supraclavicular lymph node metastasis. (6) Absence of severe lung, heart, or liver disorder. The exclusion criteria are (1) Diagnosis of esophageal fistula with accompanying esophageal stent implantation. (2) Receipt of low-dose palliative radiotherapy. (3) Receipt of preoperative or postoperative adjuvant radiotherapy. (4) Poor visualization quality on CT images.

This study collected the medical records data of ESCC patients over 75 who underwent radical radiotherapy at the Fourth Affiliated Hospital of Hebei Medical University between January 2017 and December 2019, and 130 eligible patients were enrolled in the study, with age range 75–90. The clinical stage was determined based on the American Joint Committee on Cancer (AJCC)/Union for International Cancer Control (UICC) classification scheme, 8th edition [14].

#### Treatment

89 patients among the subjects accepted radiotherapy alone, 28 received concurrent chemoradiotherapy, and 13 received a sequential chemoradiotherapy scheme. Three-dimensional conformal or intensity-modulated radiotherapy was used to carry out all treatment plans. The protocols should be followed regarding dose restrictions for the organs at risk and the definition of the radiation target volume. The total group’s Planning Target Volume (PTV) and Gross Tumor Volume (GTV) prescription radiation doses ranged from 50.0 to 64.0 Gy (median 60 Gy). PTV was given 1.8–2.0 Gy/fraction, while GTV was given 1.95–2.15 Gy/fraction, 5 times weekly. The physiotherapist completed the treatment plan as needed, and a senior physician approved it. The following was the specific chemotherapy treatment scheme [5]: TS-1, cisplatin combined with paclitaxel, and cisplatin combined with 5-fluorouracil. The final choice of the chemotherapy treatment plan was mainly due to the results of the expert decision and their treatment intention. Among the concurrent chemotherapy patients, 22 patients chose T-S1, 6 patients chose the TP scheme (paclitaxel combined with cisplatin), and among the sequential chemotherapy patients, 7 patients received the TP scheme, 4 patients chose TS-1, and 2 patients chose the FP scheme (5-fluorouracil combined with cisplatin).

### Image processing

The CT images for each patient were reviewed using itk-snap software (http://www.itksnap.org). A radiation therapist with 15-year experience in esophageal cancer (EC) imaging (A.D.Z.) reviewed all the image and delineated the outline of esophageal cancer layer by layer, and the air tissue in the esophagus is removed in the pre-treatment contrast-enhancement CT images. To evaluate inter-class agreement, a total of 20 patients were randomly selected from the entire cohort, and independent segmentation was performed by an radiologist with 15-year experience (Y.L.). Radiomics parameter extraction and image pre-processing were performed using the pyradiomics package (version 2.12; https://pyradiomics.readthedocs.io/en/2.1.2/). After normalization, resampling, and quantization in pre-processing, quantitative features based on the original image were derived from the Region of Interest of each patient. The extraction of features encompasses various categories, namely first-order statistics (first order), shape eigenvalues (shape), and texture features, which include gray level co-occurrence matrix, gray level run length matrix, gray level region size matrix, gray level difference co-occurrence matrix, and neighborhood gray level difference matrix. Clinical risk factors included as below: ECOG, age, sex, history of alcohol and tobacco, family history, length of the tumor, location of the tumor, and volume of the tumor, T stage, N stage, supraclavicular lymph node, TNM stage, PTV dose, GTV dose, whether received chemotherapy, maximal wall thickness (MWT) before RT, node size (NS) before RT.

### Model establishment

To enhance the accuracy and stability of the model, we optimized algorithms in data standardization, dimensionality reduction, and feature value screening. Various approaches were compared during the modeling process. For data standardization, we compared algorithms such as normalization to a unit, normalization to 0-center, and normalization to a unit with 0-center. Regarding dimensionality reduction, the effectiveness of principal component analysis (PCA) and Pearson correlation coefficients (PCC) methods were evaluated. In the feature screening stage, we compared the impact of multivariate analysis of variance (ANOVA), recursive feature elimination, and Relief methods on the model. The best combination scheme was then determined to establish the model. Finally, 10 classifiers including support vector machine, linear discriminant analysis, logistics regression, naive Bayes, etc. were compared. The whole training process was as follows: Firstly, all data sets were divided into training sets and test sets according to a ratio of 7:3, and then a fivefold cross-validation method was used to train the model on the training set. During training, the training data set was randomly divided into five subsets (folds) of approximately equal size. Secondly, the model was sequentially trained on four folds and validated on the remaining one, rotating until each fold has served as the validation set. In each fold, the model’s performance metrics (like accuracy, area under curve) were recorded. After completing the above steps for each fold, the performance metrics were averaged across all five folds to obtain a single estimation of model performance. Finally, the established model’s performance was evaluated with the initially generated test set.

**Fig. 1:**
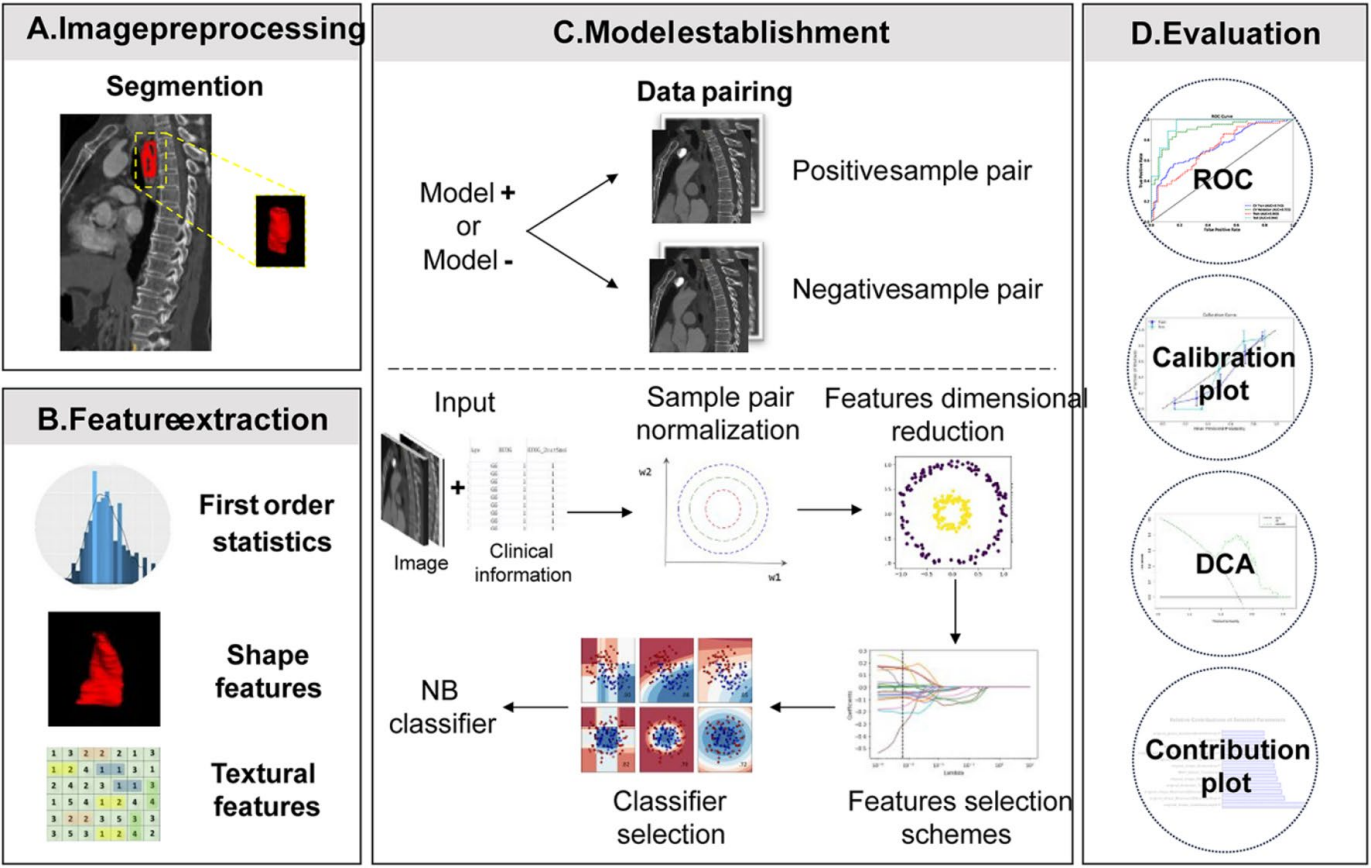
The flowchart of data preprocessing and model establishing. **A** Manual delineation of the esophageal cancer and 3D view; **B** The extraction of radiomics eigenvalues, including the first-order eigenvalues, shape and texture eigenvalues; **C** Sample-data paring and the establishment of the model; **D** Evaluation of the model

To enhance the accuracy and robustness of the model within a small sample set, we employed a pairwise machine learning analysis based on metric learning. This method relied on assessing the similarity between typical cases (templates) and other cases to predict LR following RT. In this analysis, seven representative cases were selected, comprising both experienced and non-experienced LR instances. Subsequently, these cases were paired with other samples to calculate the distance metrics. The pairs among the same group were called “positive pairs,” and the pairs among different groups called “negative pairs” (Formula 1). Finally, according to the classification results of positive and negative pairs and the label categories of the template, the final sample category was confirmed by a voting scheme (Formula 2 and 3).

1 $${\overrightarrow{\text{V}}}_{\text{P}(\text{N})\text{P}}= {\overrightarrow{\text{M}}}_{\text{I}}-{\overrightarrow{\text{N}}}_{\text{i}} \left(\text{I }\in \{\text{1,2},3,...7\};\text{ i}=1, 2, 3\dots \dots \text{n}\right)$$

2 $${\text{P}}_{\text{i}}=\text{Avg}\left({\text{P}}_{\text{PPi}}+(1-{\text{P}}_{\text{NPi}})\right) (\text{i}=1,\text{ 2,3}\dots \dots \text{n})$$

3 $${\text{N}}_{\text{i}}=\left\{\begin{array}{c} if {\text{P}}_{\text{i}} <0.5 and {\text{M}}_{\text{I }}\in 1(0) , then {\text{N}}_{\text{i }}\in 0(1)\\ if {\text{P}}_{\text{i}} >0.5 and {\text{M}}_{\text{I }}\in 1(0) , then {\text{N}}_{\text{i }}\in 1(0)\end{array}\right.$$

where, ${\overrightarrow{\text{M}}}_{\text{I}}$ and ${\overrightarrow{\text{N}}}_{\text{i}}$ represent eigenvalue vector of the $I$th template and the $i$th sample, ${\overrightarrow{\text{V}}}_{\text{PP}}$ represents eigenvalue vector of the positive pair, ${\overrightarrow{\text{V}}}_{\text{NP}}$ represents eigenvalue vector of the negative pair, ${\text{P}}_{\text{PPi}}$ and ${\text{P}}_{\text{NPi}}$ represent predicted probability of positive pair and negative pair, ${\text{P}}_{\text{i}}$ represents to the average probability that a sample eventually belongs to the positive sample pair, ${\text{M}}_{\text{I}}$ represents the label of a template, ${\text{N}}_{\text{i}}$ represents the class attribute of a sample. According to the diagnostic performance, the optimal model was determined. The whole modeling process was shown in Fig. 1.

### Statistical analysis

To determine the accuracy and the repeatability of the delineated tumor volume, the average Dice and the inter-class correlation coefficient (ICC) about the volume, surface area, maximum diameter length, minimum diameter length and other morphological indicators of the two delineated tumors were calculated respectively. The baseline differences in clinical characteristics between the training set and testing set were evaluated using statistical tests such as the Chi-squared test, Fisher’s exact test, or Mann–Whitney U-test. Additionally, a decision curve analysis (DCA) was performed on the testing dataset to assess the clinical utility of the model, quantifying the net benefits across various threshold probabilities. To evaluate the model fit, the goodness of fit was assessed using the Hosmer–Lemeshow test. The performance of the model was assessed via the area under the receiver operating characteristic curve (AUC). A p value of < 0.05 was considered statistically significant. The statistical analysis is performed using the keras, pingouin and pROC packages based on python 3.10 and R language (V4.2.1) respectively.

## Results

### The general clinical characteristics of patients

This study included 130 elderly patients in total. All patients had been followed for more than three years as of March 1, 2023, with a median follow-up of 57 months. The overall rate of follow-up was 96.92%. According to the 7:3 ratio, the total cases are randomly assigned into training group and testing group. Table 1 presents the characteristics of ESCC patients in two groups, revealing no significant differences between them. At the follow-up date, a total of 64 patients (49.23%) had encountered LR. Among these, 45 patients (49.45%) belonged to the training group, while 19 patients (48.72%) were from the testing group.

**Table 1:** Characteristics of patients in training cohort and testing cohort

Characteristics	Training cohort n = 91	Testing cohort n = 39	P
ECOG			0.266
0–1	22(24.2%)	6(15.4%)	
2	69(75.8%)	33(84.6%)	
Gender			0.561
Male	54(59.3%)	21(53.8%)	
Female	37(40.7%)	18(46.2%)	
Smoking and Alcohol			0.238
Yes	53(58.2%)	27(69.2%)	
No	38(41.8%)	12(30.8%)	
Family History			0.641
Yes	79(86.8%)	35(89.7%)	
No	12(13.2%)	4(10.3%)	
Location			0.116
Cervical	5(5.5%)	2(5.1%)	
Upper	32(35.2%)	8(20.5%)	
Middle	43(47.3%)	22(56.4%)	
Lower	11(12.1%)	7(17.9%)	
T Stage			0.446
T2	11(12.1%)	2(5.1%)	
T3	45(49.5%)	2(71.8%)	
T4a	22(24.2%)	4(10.3%)	
T4b	13(14.3%)	5(12.8%)	
N Stage			0.439
N0	5(5.5%)	2(5.1%)	
N1	25(27.5%)	6(15.4%)	
N2	35(38.5%)	20(51.3%)	
N3	26(28.6%)	11(28.2%)	
Supraclavicular LN			0.765
Yes	80(87.9%)	35(89.7%)	
No	11(12.1%)	4(10.3%)	
TNM stage			0.599
II	8(8.8%)	3(7.7%)	
III	33(36.3%)	17(43.6%)	
IVA	39(42.9%)	15(38.5%)	
IVB	11(12.1%)	4(10.3%)	
MWT_before_RT(cm)	1.99 ± 0.57	2.08 ± 0.53	0.421
NS_before_RT(cm)	1.21 ± 0.66	1.11 ± 0.45	0.617
GTV dose(Gy)			0.154
50–60	30(33.0%)	18(46.2%)	
60	38(41.8%)	14(35.9%)	
60–66	23(25.3%)	7(17.9%)	
PTV dose(Gy)			0.352
50–60	48(52.7%)	25(64.1%)	
60	30(33.0%)	8(20.5%)	
60–66	13(14.3%)	6(15.4%)	
Chemotherapy			0.773
Yes	28(30.77%)	13(33.33%)	
No	63(69.23%)	26(66.67%)	
LR			0.939
Yes	45(49.45%)	19(48.72%)	
No	46(50.55%)	20(51.28%)	

### Model construction

**Fig. 2:**
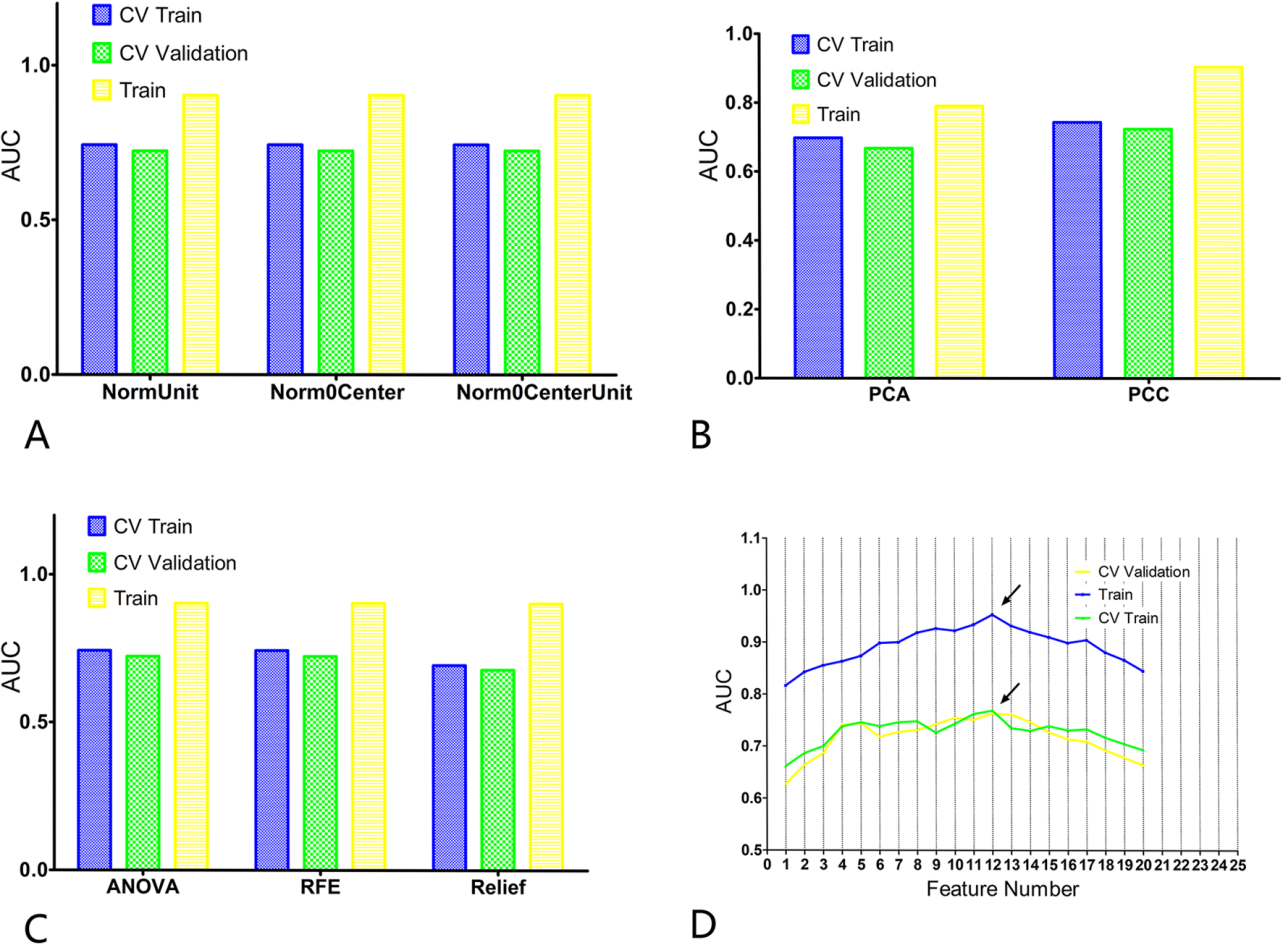
The modeling process of the model. **A** Normalize to unit with 0-center demonstrated the best performance than other schemes in data normalization. **B** PCC scheme demonstrated better performance than the PCA scheme in features dimensionality reduction. **C** ANOVA scheme demonstrated the best performance than other two schemes in the stage of features screening. **D** The model demonstrates best performance when 12 eigenvalues were employed. CV Train: Result under the training data via a fivefold cross validation method; CV Validation: Result under the validation data via a fivefold cross validation method; Train: result using all training data

**Fig. 3:**
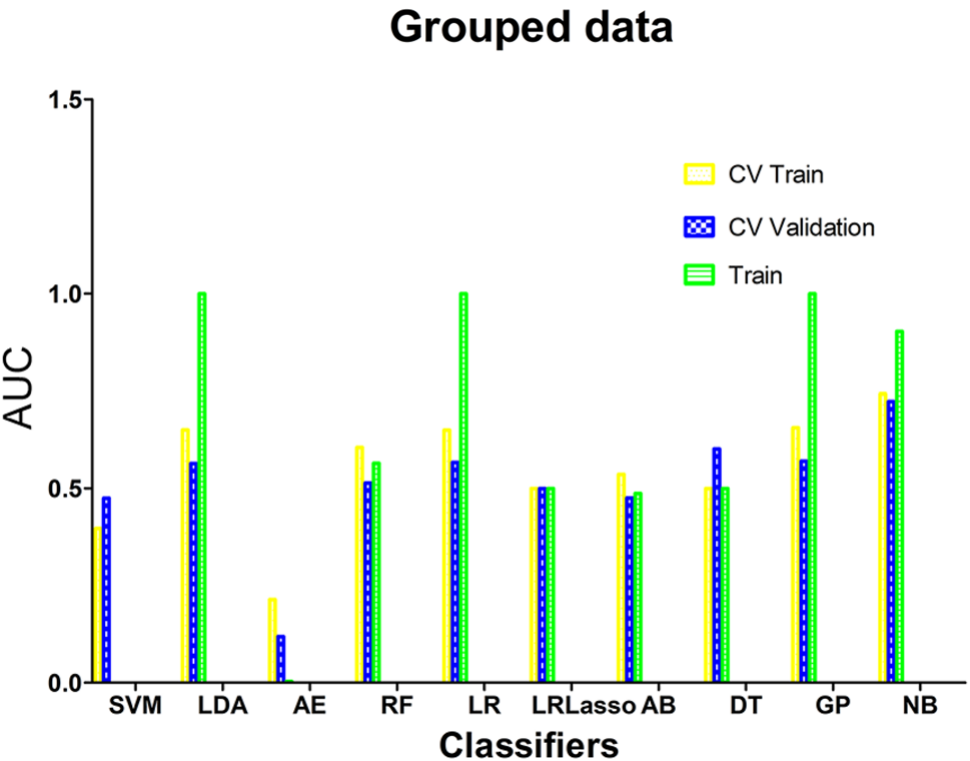
The comparison of diagnostic performance among different classifiers

The calculated results showed that the mean Dice was 0.891 ± 0.452 and ICCs of some important morphological factors were summarized as in Table 2, which indicating good reproducibility. After optimization, an optimal intelligent diagnostic model, named pair-wise native bayes (pNB), was selected which including a normalize to unit with 0-center data normalization scheme, a PCC features dimensionality reduction scheme, an ANOVA features screening scheme, 12 eigenvalues as input and an optimized native bayes classifier as the final binary classifier (Fig. 2, Supplementary Table 1). After evaluating, the pNB model demonstrates best performance in the training, verification and testing dataset for predicting LR in elderly patients with ESCC (Fig. 3).

**Table 2:** The summary of ICCs for some important morphological factors

Feature Name	ICC (95% CI)		F	P
Least axis length	0.976	[0.81–0.99]	116.890	< 0.001
Major axis length	0.992	[0.97–1.00]	245.268	< 0.001
Maximum 2D diameter column	0.986	[0.94–1.00]	178.457	< 0.001
Maximum 2D diameter row	0.989	[0.96–1.00]	203.656	< 0.001
Maximum 2D diameter slice	0.914	[0.54–0.98]	34.941	< 0.001
Maximum 3D diameter	0.990	[0.96–1.00]	230.047	< 0.001
Mesh volume	0.973	[0.71–0.99]	141.526	< 0.001
Minor axis length	0.967	[0.29–0.99]	204.080	< 0.001
Sphericity	0.768	[0.33–0.94]	7.376	< 0.001
Surface area	0.984	[0.94–1.00]	136.733	< 0.001
Surface volume ratio	0.865	[0.46–0.97]	19.386	< 0.001
Voxel volume	0.972	[0.71–0.99]	139.226	< 0.001

### Model performance

**Fig. 4:**
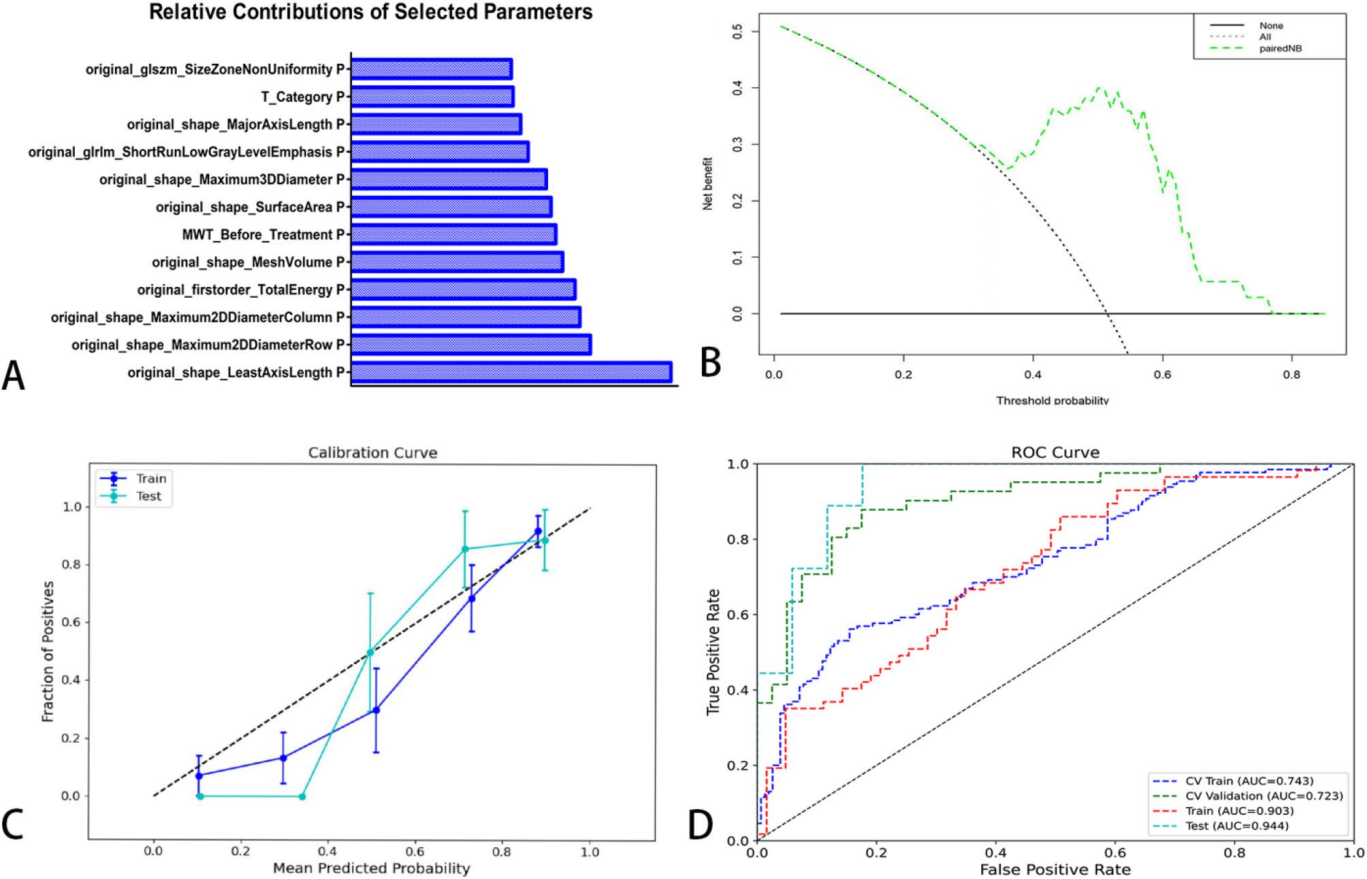
The diagnostic performance and stability of the paired-wise NB model. **A** The selected eigenvalues and their contributions in the model after optimization. **B** DCA of the model; **C** The calibration curve of the model in the training set and testing set; **D** The AUC of ROC on CV training, CV validation, training and testing data. Test: result using all testing data

A total of twelve features were included in the modeling process, comprising ten radiomics features and two clinical factors. The selected eigenvalues and their respective contributions in the optimized model are visually represented in Fig. 4A. The model was evaluated from the following three dimensions: diagnostic performance, calibration, and clinical validity. Based on the model created in this study, the DCA revealed that, based on the paired-wise NB model, when the threshold probability is less than 80% or higher than 40%, then more benefit can be obtained than a biopsy all, or biopsy none scheme (Fig. 4B). The calibration curves indicate that the model exhibited excellent goodness of fit and stability (P > 0.05) (Fig. 4C). The area under the curve (AUC) of the training group and testing group were 0.903 (0.829–0.958) and 0.944 (0.849–1.000), respectively, and the corresponding accuracies were 0.852 and 0.914. The sensitivity and specificity in the training group and testing group were 0878, 0.825 and 1.000, 0.824. The positive predictive value and negative predictive value were 0.837 and 0.868 (training group), 0.857 and 1.000 (testing group), respectively (Fig. 4D) (Supplementary Table 2). These findings show that this model could forecast LR precisely in elderly patients with ESCC who underwent RT.

### Establishment of automated modeling system and automated diagnostic system

In this experiment, we established an automatic modeling system based on the open-source software FAE using metric learning. This system can automatically perform data pairing, standardization of data pairs, dimensionality reduction, feature selection, and classifier selection for modeling.

**Fig. 5:**
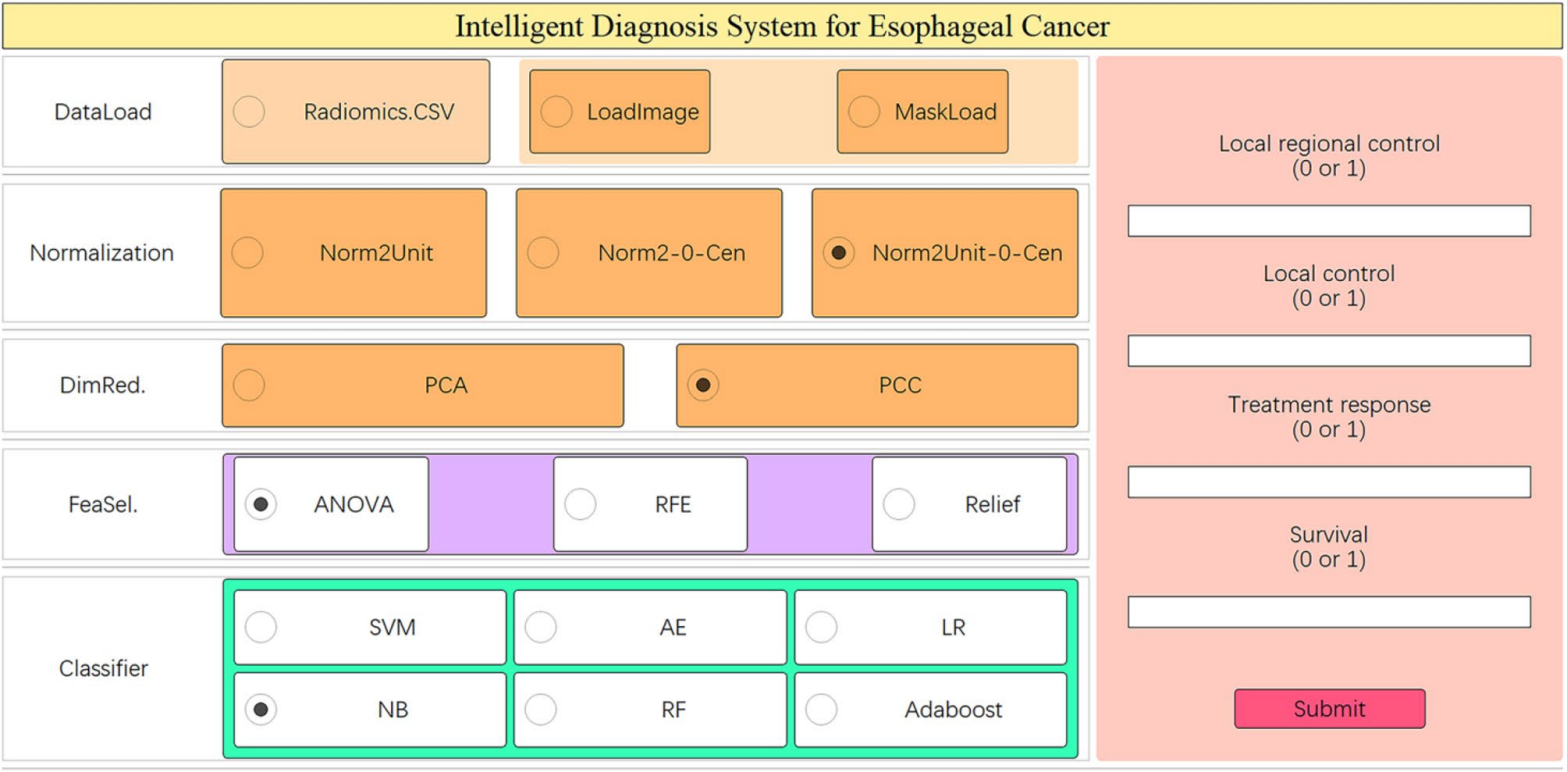
The automated graphical user interface (GUI) diagnostic system

Additionally, to facilitate clinical application of the well-modeled models by medical professionals, we developed a graphical user interface (GUI) diagnostic system (Fig. 5), which can be downloaded from Uniform Resource Locator (https://github.com/ComputerVersion/PairedML). Unlike the web-based version of an automated diagnostic system, our diagnostic system operates as a standalone version, taking into consideration both data privacy concerns in hospitals and limitations in internet speed in certain regions. Built upon preliminary modeling results, this interface-based system allows the selection of appropriate preprocessing methods and classifiers, along with the choice of corresponding pre-trained weight values. By inputting relevant parameter values, the system can automatically provide diagnostic outcomes. It supports two types of inputs: images and radiomics features and enables the prediction of various tasks in esophageal cancer diagnosis.

## Discussion

In recent years, the development of radiomics and machine learning techniques has shown great potential in clinical diagnosis and treatment. Several studies have highlighted the use of machine learning techniques, based on radiomics and clinical information, for predicting treatment response and prognosis in patients with esophageal cancer [9–11]. Traditional machine learning models exhibit a diagnostic efficiency of around 0.7 [9, 10, 15]. We believe the low predictive accuracy may be due to several reasons: first, traditional machine learning methods have limited ability to explore information, leading to difficulty in distinguishing between typical and atypical cases. Second, there is often an imbalance between categories, particularly between cases with positive and negative treatment outcomes, with fewer cases typically having poor outcomes. Third, the limited amount of data available restricts the models’ accuracy and generalizability, making it difficult to achieve higher performance. Given these challenges, we propose a paired-sample machine learning model algorithm based on metric learning. This approach significantly improves predictive accuracy and demonstrates strong generalizability.

The ability of model generalization refers to the performance of a model on unseen data, which indicates the model’s adaptability to new data. How to improve the generalization ability of a model is a highly focused issue in the field of machine learning, especially in medical data analysis, due to the lack of precise labeled data and the high cost of annotation [16]. Currently, there are several commonly used methods to improve model generalization ability: Transfer learning [17–21]. The advantage of this method is its simplicity and ease of implementation, but it requires some similarities between the source domain and the target domain. Meta-learning [22, 23] is also a commonly used method in addressing the small sample classification tasks. Metric-based meta-learning methods [24] utilize metrics to express the correlation between two samples. Metrics measure the relationship between two samples based on the distance calculated in the space, where closer points indicate a higher likelihood of belonging to the same category. Metric learning is a machine learning approach which captures the underlying structure of the data [25]. The objective of metric learning is to optimize a distance metric learning objective function that incorporates desired properties. By learning a distance metric that is tailored to the underlying structure of the data, metric learning algorithms can often achieve better performance than traditional algorithms that rely on fixed distance metrics such as Euclidean distance. Another advantage of metric learning is its generalizability. Metric learning algorithms can learn a distance metric that is generalizable to new data, even if the data has not been seen during training. Overall, the theoretical foundation and advantages of metric learning make it a powerful and versatile tool in clinical applications, particularly in the field of medical image analysis and diagnosis [26, 27]. In this experiment, we developed a model analysis algorithm that leverages future data features and the strengths of metric learning. We tested and applied this tool on a small sample set.

The research paper aimed to develop an intelligent model for accurately predicting LR in elderly patients with ESCC who underwent RT. Following optimization, an optimal diagnostic model called pair-wise native Bayes was selected. The pNB model incorporated various components, including data normalization, feature dimensionality reduction, feature screening, and an optimized native Bayes classifier (Fig. 2, Table 2). Ten radiomics features and two clinical factors were finally chosen for modeling in the study and the results as depicted in Fig. 4A visually presented the selected eigenvalues and their respective contributions in the optimized model. The two clinical factors are: MWT before Treatment and T stage. According to Li’s study [28], the maximum esophageal wall thickness was a significant factor affecting OS in ESCC patients. Samely, in this study, MWT and T stage were also important factors affecting LR in elderly patients. In literatures’ report, the shape features were noted to be the most robust and stable. The present study finds that the most important feature was original shape Least Axis Length, which was also a shape-related feature [29–31]. The first-order feature is a function of the gray level of the image, reflecting the distribution of voxel gray intensity in the region. Energy represents the extent of voxel value magnitudes within an image, whereby a higher value signifies an augmented sum of squared voxel values [32]. The Total Energy feature corresponds to the Energy value, which has been adjusted according to the voxel volume in cubic mm. In this experiment, we found that the total energy has a high predictive value for esophageal cancer radiotherapy, which is consistent with our previous knowledge that the degree of enhancement and size of the lesion has a high diagnostic value [28, 33]. The evaluation of the model was conducted from three dimensions: diagnostic performance, calibration, and clinical validity. These results demonstrate that the developed model can accurately forecast LR in elderly patients with ESCC who underwent RT. The findings suggest the potential clinical utility of this pNB model for improving decision-making and patient management in this population. The successful development and performance of the pNB model have significant implications for clinical practice. Accurate prediction of LR can aid in treatment planning and decision-making for elderly patients with ESCC undergoing therapy. Overall, the findings of this study contribute to the advancement of predictive modeling in ESCC and hold promise for improving patient outcomes and personalized treatment strategies. In this study, we have also developed a standalone application for this model, as illustrated in Fig. 5. In this experiment, the rationale for choosing a standalone version of the automated diagnostic system, rather than a web-based version, is as follows: (1) The standalone version offers greater convenience by not requiring users to upload patient data to the internet, thus better safeguarding patient privacy; (2) The standalone version is not impacted by varying internet speeds across different regions; and (3) The standalone version is more conducive to commercialization and broader dissemination. This study possesses distinct advantages compared to previous investigations. To the best of our knowledge, this marks the inaugural machine learning model devised for prognosticating LR among elderly patients diagnosed with ESCC who have undergone RT. Notably, our model exhibits robustness when applied to small sample datasets, yielding accurate predictive performance.

This study has some limitations. The first is its reliance on manual image segmentation for intelligent image analysis, resulting in time-consuming procedures. However, with advancements in technology, automated intelligent image recognition and segmentation may mitigate labor requirements and time consumption. Another limitation is the retrospective nature of this single-center study. Given the differences in the incidence of pathological types of esophageal cancer in different regions and the resulting varying treatment responses [34, 35], further research and validation are necessary to confirm the model’s performance in larger and more diverse patient populations. In our experience, this can be improved by including templates from different samples when generating paired samples. Additionally, various methods for radiomics analysis exist, and the obtained results may vary, necessitating further comparisons and discussions. The stand-alone application for this model will allow readers to further validation using external datasets and refine the model based on our solution for wider adoption in clinical practice, and assessing the model’s performance against other existing predictive models would provide valuable insights into its comparative effectiveness.

## Conclusion

The pairwise NB model, based on pre-treatment enhanced chest CT-based radiomics and clinical factors, accurately predicts LR in elderly patients with ESCC. The standalone application facilitates external validation and refinement, promoting wider clinical adoption.

## Supplementary Information

Below is the link to the electronic supplementary material.

Supplementary file1 (DOCX 14 KB)

## Data Availability

The datasets generated during and/or analyzed in the current study are available from the corresponding authors upon reasonable request.

## Abbreviations

EC: Esophageal cancer
RT: Radiotherapy
CRT: Chemoradiotherapy
LR: Locoregional Recurrence
NB: Naïve Bayes
ESCC: Esophageal Squamous Cell Carcinoma
ECOG: Eastern Cooperative Oncology Group
PCA: Principal component analysis
PCC: Pearson correlation coefficients
ICC: Inter-class correlation coefficient
AUC: Area under the curve
ROC: Receiver Operating Characteristic
DCA: Decision curve analysis
MWT: Maximal wall thickness
NS: Node size
PTV: Planning Target Volume
GTV: Gross Tumor Volume

## References

[1] Sung H, Ferlay J, Siegel RL, et al. Global Cancer Statistics 2020: GLOBOCAN Estimates of Incidence and Mortality Worldwide for 36 Cancers in 185 Countries. CA Cancer J Clin. 2021. 71(3): 209-249. 33538338 10.3322/caac.21660

[2] Haibo Qiu, Sumei Cao, Ruihua Xu. Cancer incidence, mortality, and burden in China: a time-trend analysis and comparison with the United States and United Kingdom based on the global epidemiological data released in 2020. Cancer Commun (Lond). 2021; 41(10):1037-1048. 34288593 10.1002/cac2.12197 PMC8504144

[3] Zhang Andu, Han Chun, Lan Kuntian, et al. Analysis of age and prognosis in patients with esophageal squamous cell carcinoma after 3DCRT. Chinese Journal of Radiation Oncology, 2014, 23(5), 392-395.

[4] Vlacich G, Samson PP, Perkins SM, et al. Treatment utilization and outcomes in elderly patients with locally advanced esophageal carcinoma: a review of the National Cancer Database. Cancer Med. 2017. 6(12): 2886-2896. 29139215 10.1002/cam4.1250 PMC5727236

[5] Li-Li Zhu, Ling Yuan, Hui Wang, et al. A Meta-Analysis of Concurrent Chemoradiotherapy for Advanced Esophageal Cancer. PLoS One. 2015; 10(6): e0128616. 26046353 10.1371/journal.pone.0128616 PMC4457836

[6] Jin X, Zheng X, Chen D, et al. Prediction of response after chemoradiation for esophageal cancer using a combination of dosimetry and CT radiomics. Eur Radiol. 2019. 29(11): 6080-6088. 31028447 10.1007/s00330-019-06193-w

[7] Chetan MR, Gleeson FV. Radiomics in predicting treatment response in non-small-cell lung cancer: current status, challenges and future perspectives. Eur Radiol. 2021. 31(2): 1049-1058. 32809167 10.1007/s00330-020-07141-9 PMC7813733

[8] Wong PK, Chan IN, Yan HM, et al. Deep learning based radiomics for gastrointestinal cancer diagnosis and treatment: A minireview. World J Gastroenterol. 2022. 28(45): 6363-6379. 36533112 10.3748/wjg.v28.i45.6363 PMC9753055

[9] Hu Y, Xie C, Yang H, et al. Computed tomography-based deep-learning prediction of neoadjuvant chemoradiotherapy treatment response in esophageal squamous cell carcinoma. Radiother Oncol. 2021. 154: 6-13. 32941954 10.1016/j.radonc.2020.09.014

[10] Gong J, Zhang W, Huang W, et al. CT-based radiomics nomogram may predict local recurrence-free survival in esophageal cancer patients receiving definitive chemoradiation or radiotherapy: A multicenter study. Radiother Oncol. 2022. 174: 8-15. 35750106 10.1016/j.radonc.2022.06.010

[11] Ruben T H M Larue, Remy Klaassen, Arthur Jochems, et al. Pre-treatment CT radiomics to predict 3-year overall survival following chemoradiotherapy of esophageal cancer. Acta Oncol. 2018; 57(11):1475-1481. 30067421 10.1080/0284186X.2018.1486039

[12] Yan-Ling Li, Li-Ze Wang, Qing-Lei Shi, et al. CT Radiomics for Predicting Pathological Complete Response of Axillary Lymph Nodes in Breast Cancer. Oncologist. 2023;28(4):e183-e190. 36802345 10.1093/oncolo/oyad010 PMC10078899

[13] Chen W, Zheng R, Baade PD, et al. Cancer statistics in China, 2015. CA Cancer J Clin. 2016. 66(2): 115-32. 26808342 10.3322/caac.21338

[14] Rice TW, Ishwaran H, Ferguson MK, et al. Cancer of the Esophagus and Esophagogastric Junction: An Eighth Edition Staging Primer. J Thorac Oncol. 2017. 12(1): 36-42. 27810391 10.1016/j.jtho.2016.10.016 PMC5591443

[15] Luo HS, Chen YY, Huang WZ, et al. Development and validation of a radiomics-based model to predict local progression-free survival after chemo-radiotherapy in patients with esophageal squamous cell cancer. Radiat Oncol. 2021. 16(1): 201. 34641928 10.1186/s13014-021-01925-z PMC8513312

[16] Rajpurkar P, Joshi A, Pareek A, et al. CheXpedition: investigating generalization challenges for translation of chest x-ray algorithms to the clinical setting. arXiv preprint arXiv:200211379 2020.

[17] Yosinski J, Clune J, Bengio Y, et al. How transferable are features in deep neural networks? Advances in neural information processing systems 2014, 27.

[18] Oquab M, Bottou L, Laptev I, et al. Learning and transferring mid-level image representations using convolutional neural networks. In: Proceedings of the IEEE conference on computer vision and pattern recognition: 2014. 1717–1724.

[19] Yao Y, Doretto G. Boosting for transfer learning with multiple sources. In: 2010 IEEE computer society conference on computer vision and pattern recognition: 2010. IEEE: 1855–1862.

[20] Pan SJ, Yang Q. A survey on transfer learning. IEEE Transactions on knowledge and data engineering 2010, 22(10):1345-1359.

[21] Zadrozny B. Learning and evaluating classifiers under sample selection bias. In: Proceedings of the twenty-first international conference on Machine learning: 2004. 114.

[22] Huisman M, Van Rijn JN, Plaat A. A survey of deep meta-learning. Artificial Intelligence Review 2021, 54(6):4483-4541.

[23] Lee K, Maji S, Ravichandran A, et al. Meta-learning with differentiable convex optimization. In: Proceedings of the IEEE/CVF conference on computer vision and pattern recognition: 2019. 10657–10665.

[24] Sung F, Yang Y, Zhang L, et al. Learning to compare: Relation network for few-shot learning. In: Proceedings of the IEEE conference on computer vision and pattern recognition: 2018. 1199–1208.

[25] Lee S. Introduction and Perspective of Deep Metric Learning. Available at SSRN 4320050 2023.

[26] Nwanosike EM, Conway BR, Merchant HA, et al. Potential applications and performance of machine learning techniques and algorithms in clinical practice: a systematic review. International Journal of Medical Informatics 2022, 159:104679. 34990939 10.1016/j.ijmedinf.2021.104679

[27] Chen X, Wang X, Zhang K, et al. Recent advances and clinical applications of deep learning in medical image analysis. Medical Image Analysis 2022, 79:102444. 35472844 10.1016/j.media.2022.102444 PMC9156578

[28] Tingting Li, Xiaobin Fu, Lihua Xiao, et al. The long-term impact of tumor burden in pT3N0M0 esophageal squamous cell carcinoma. Medicine (Baltimore). 2019; 98(42): e17637. 31626150 10.1097/MD.0000000000017637 PMC6824748

[29] Mohammed R. S. Sunoqrot, Kirsten M. Selnæs, Elise Sandsmark, et al. A Quality Control System for Automated Prostate Segmentation on T2-Weighted MRI. Diagnostics (Basel). 2020; 10(9): 714. 32961895 10.3390/diagnostics10090714 PMC7555425

[30] Yang Zhang, Ching-Chung Ko, Jeon-Hor Chen, et al. Radiomics Approach for Prediction of Recurrence in Non-Functioning Pituitary Macroadenomas. Front Oncol. 2020; 10: 590083. 33392084 10.3389/fonc.2020.590083 PMC7775655

[31] Fiset S, Welch ML, Weiss J, et al. Repeatability and reproducibility of MRI-based radiomic features in cervical cancer. Radiother Oncol (2019) 135:107–14. 31015155 10.1016/j.radonc.2019.03.001

[32] Daisuke Kawahara, Xueyan Tang, Chung K. Lee, et al. Predicting the Local Response of Metastatic Brain Tumor to Gamma Knife Radiosurgery by Radiomics With a Machine Learning Method. Front Oncol. 2020; 10: 569461. 33505904 10.3389/fonc.2020.569461 PMC7832385

[33] Djuric-Stefanovic A, Jankovic A, Saponjski D, et al. Analyzing the post-contrast attenuation of the esophageal wall on routine contrast-enhanced MDCT examination can improve the diagnostic accuracy in response evaluation of the squamous cell esophageal carcinoma to neoadjuvant chemoradiotherapy in comparison with the esophageal wall thickness. Abdom Radiol (NY), 2019, 44(5):1722-1733. 30758534 10.1007/s00261-019-01911-w

[34] Eileen Morgan, Isabelle Soerjomataram, Harriet Rumgay, et al. The Global Landscape of Esophageal Squamous Cell Carcinoma and Esophageal Adenocarcinoma Incidence and Mortality in 2020 and Projections to 2040: New Estimates From GLOBOCAN 2020. Gastroenterology. 2022, 163(3):649-658.e2. 35671803 10.1053/j.gastro.2022.05.054

[35] Kazuto Harada, Jane E Rogers, Masaaki Iwatsuki, et al. Recent advances in treating oesophageal cancer. F1000Res. 2020. 9:F1000 Faculty Rev-1189. 10.12688/f1000research.22926.1 33042518 10.12688/f1000research.22926.1 PMC7531047

