# Supplementary material for: Pairwise machine learning-based automatic diagnostic platform utilizing CT images and clinical information for predicting radiotherapy locoregional recurrence in elderly esophageal cancer patients
Source: Abdom Radiol (NY). 2024 Jun 4;49(11):4151–61. doi: 10.1007/s00261-024-04377-7 (PMC11519085; doi:10.1007/s00261-024-04377-7)
Supplement: Supplementary file 1 — Supplementary file1 (DOCX 14 KB) [file 261_2024_4377_MOESM1_ESM.docx]

**Supplementary Table 1 Classifier Comparision (AUC)**

|  | SVM | LDA | AE | RF | LR | LR Lasso | AB | DT | GP | NB |
| --- | --- | --- | --- | --- | --- | --- | --- | --- | --- | --- |
| CV  Train | 0.397 | 0.651 | 0.214 | 0.605 | 0.650 | 0.500 | 0.536 | 0.500 | 0.656 | 0.743 |
| CV Validation | 0.475 | 0.564 | 0.119 | 0.514 | 0.567 | 0.500 | 0.475 | 0.602 | 0.570 | 0.723 |
| Train | 0 | 1.000 | 0.003 | 0.565 | 1.000 | 0.500 | 0.487 | 0.500 | 1.000 | 0.903 |

SVM:Support Vector Machine; LDA:Latent Dirichlet Allocation; AE:Auto-Encoder; RF:Random Forest; LR: Logistic Regression; LR Lasso: Logistic Regression Lasso; AB: Alpha-Beta; DT: Decision Tree; GP: Gaussian process; NB: naïve bayes

**Supplementary Table 2 Predictive performance of model**

|  | accuracy | AUC(95%CI) | NPV | PPV | sensitivity | specificity |
| --- | --- | --- | --- | --- | --- | --- |
| Train cohort | 0.8519 | 0.903(0.8290-0.9579) | 0.8684 | 0.8372 | 0.878 | 0.825 |
| Testing cohort | 0.9143 | 0.9444(0.8487-1.0000) | 1 | 0.8571 | 1 | 0.8235 |
| CV Train | 0.7158 | 0.7427(0.6839-0.8003) | 0.6968 | 0.7526 | 0.5615 | 0.8452 |
| CV Test | 0.6667 | 0.7232(0.6319-0.8062) | 0.7949 | 0.6049 | 0.8596 | 0.4921 |

NPV:negative predictive value; PPV:positive predictive value
